# Supplementary material for: Vitamin D Status, Vitamin D Receptor Polymorphisms, and the Risk of Incident Rosacea: Insights from Mendelian Randomization and Cohort Study in the UK Biobank
Source: Nutrients. 2023 Aug 30;15(17):3803. doi: 10.3390/nu15173803 (PMC10489658; doi:10.3390/nu15173803)
Supplement: Supplementary file 1 [file nutrients-15-03803-s001.zip › nutrients-2591038-supplementary.pdf]

Supplementary Materials

eMethods

Table S1. Serum 25(OH)D concentrations according to different VDR genotypes among patients with rosacea in the UK Biobank.

Table S2. Adjusted HRs and 95% CI for VDR Polymorphisms with rosacea in the UK Biobank study.

Table S3. The joint associations of serum 25(OH)D and genetic variants in rs731236 (*TaqI*) with risk of rosacea.

Table S4. Interaction effect between serum 25OHD and sex, age, body mass index, sleeplessness, 25OHD supplement, and VDR Polymorphisms.

Table S5. Stratified analyses of the associations between serum 25(OH)D concentrations and rosacea by sex, age, BMI, sleeplessness, Physical activity level, and 25OHD supplement.

Table S6. Multivariable-adjusted HRs (95% CIs) for associations between serum 25OHD concentrations and rosacea according to different sex among participants younger than 60 years old.

Table S7. Multivariable-adjusted HRs (95% CIs) for associations between VDR Polymorphisms and rosacea according to different sex**.**

Table S8. Association of serum 25OHD with incident rosacea in participants with male and with GG allele of rs731236(*TaqI*).

Table S9. Sensitivity analysis for exclusion criteria.

Table S10. Instrumental variables used in MR analysis of the association between

Serum 25-Hydroxyvitamin D levels and rosacea.

Table S11. Mendelian randomization analyses for the association of serum 25OHD concentration with rosacea.

Table S12. Test for heterogeneity in Mendelian randomization analysis.

Table S13. Test for horizontal pleiotropy in Mendelian randomization analysis.

Figure S1. Flowchart for the Selection of the Analysed Study Sample from the UK Biobank Study.

Figure S2. Associations of serum 25(OH)D concentrations with risks of rosacea in the UK Biobank.

Figure S3. Cumulative risk curve for incident rosacea between different serum 25OHD categories.

Mendelian Randomization Analysis

Based on published genome-wide association study (GWAS) studies[30] and the FinnGen consortium[31], We performed MR Analysis using R 4.2.0 and the 'TwoSampleMR' package. GCST010144 (N=443,734, number of SNPs = 15,847,859)[30] and ebi-a-GCST90000615 (N=417,580, number of SNPs = 8,643,988)[32] data were collected as exposure variables. For rosacea, results were extracted from studies in European populations (1,195 cases and 211,139 controls, number of SNPs = 16,380,452) to generate outcome variables (ID: Finn-b-L12_ROSACEA). Statistical methods can be found in the guide (https://mrcieu.github.io/TwoSampleMR). We use *Inverse variance weighted* (IVW), *maximum likelihood* (ML), *MR-Egger regression*, *Weighted mode*, and *Weighted median* as the primary methods. Based on meta (version 6.2.1)[33] packages, we performed meta-analysis on the results of each MR analysis of the two exposed data. Then, we tested the poly-effect using MR-egger regression. The F statistic is calculated by the formula $F = \left(\frac{R^2}{1-R^2} \times \frac{(n-k-1)}{k}\right)$ (where R^2 is the proportion of the variance of the trait explained by the SNP, k is the number of Ivs, and N is the sample size of the GWAS of the SNP with the trait), which is used to quantify the strength of the instrument. A value >10 is considered sufficient. We used the TwoSampleMR R package to calculate the coefficient of determination (R^2) of exposure on genetic variants. The R^2 value was estimated using the formula $R^2 = 2 \times EAF \times (1 - EAF) \times (\beta)^2$ (where EAF is the effect allele frequency (EAF) of SNP, SD is the standard deviation, and β is the estimated effect size of SNP on the trait). Based on the online MR-power calculation tool (https://sb452.shinyapps.io/power/),[34] we calculated

the power of the MR estimates.

After removing the linkage imbalance, 92 (GCST010144) and 110 (ebi-a-GCST90000615) genetic variants ($P < 5 * 10^{-8}$, MAF ≤ 0.01, clumping window size=10,000 kb) were selected from the database as exposure tools (**Table S10**) and included them in the resulting data table (**Table S11**). The explaining rate of the total variation (R2 values) of ebi-a-GCST90000615 and GCST010144 in rosacea were 4.63% and 4.74%. The F values of ebi-a-GCST90000615 and GCST010144 were 184.2 and 240.0, excluding the possibility of weak genetic tool variables. MR-Egger regression was performed for each exposure-outcome pair to test the pleiotropy of our analysis. The results showed no significant pleiotropy ($P = 0.191$ for GCST010144; $P = 0.814$ for ebi-a-GCST90000615). The test results of heterogeneity and level pleiotropy are shown in **Tables 12 and 13**. The power of our MR analysis of ebi-a-GCST90000615 and GCST010144 were 23.1% and 22.4%.

Table S1. Serum 25(OH)D concentrations according to different VDR genotypes among patients with rosacea in the UK Biobank

	**Serum 25(OH)D, nmol/L[a]**	***P value***
**rs731236 (*TaqI*)**		
GG, N = 59,477	48.00 (33.50, 63.30)	
AG, N = 177,117	48.10 (33.60, 63.40)	0.054
AA, N = 133,615	47.90 (33.40, 63.30)	
**rs1544410 (*BsmI*)**		
TT, N = 61,293	47.80 (33.40, 63.20)	
TC, N = 178,118	48.00 (33.60, 63.40)	0.14
CC, N = 130,798	48.00 (33.50, 63.30)	
**rs7975232 (*ApaI*)**		
AA, N = 103,294	47.80 (33.40, 63.20)	
AC, N = 184,352	48.00 (33.60, 63.40)	0.028
CC, N = 82,563	48.10 (33.60, 63.40)	
**rs2228570 (*FokI*)**		
GG, N = 141,007	47.80 (33.20, 63.20)	
AG, N = 174,736	48.00 (33.70, 63.30)	<0.001
AA, N = 54,466	48.40 (34.00, 63.90)	

a. Data are presented as median (interquartile range).

Table S2. Adjusted HRs and 95% CI for VDR Polymorphisms with rosacea in the UK Biobank study

variables	No. of Person-Years	Incident rate	Incident rosacea	Unadjusted model		Fully adjusted model[a]	
				HR (95%CI)	p-value	HR (95%CI)	p-value
apal:							
CC	1091692	0.420	459 (23.7%)	Ref.	Ref.	Ref.	Ref.
AC	2438793	0.396	965 (49.8%)	0.94 [0.84;1.05]	0.285	0.94 [0.84;1.05]	0.298
AA	1365094	0.377	514 (26.5%)	0.90 [0.79;1.02]	0.085	0.90 [0.79;1.02]	0.100
				p.trend	0.225	p.trend	0.258
bsml:							
CC	1729842	0.420	726 (37.5%)	Ref.	Ref.	Ref.	Ref.
TC	2355647	0.385	907 (46.8%)	0.92 [0.83;1.01]	0.083	0.91 [0.83;1.01]	0.071
TT	810089	0.377	305 (15.7%)	0.90 [0.78;1.03]	0.110	0.89 [0.78;1.02]	0.094

Table S2. Adjusted HRs and 95% CI for VDR Polymorphisms with rosacea in the UK Biobank study

variables	No. of Person-Years	Incident rate	Incident rosacea	Unadjusted model		Fully adjusted model[a]	
				HR (95%CI)	p-value	HR (95%CI)	p-value
				p.trend	0.138	p.trend	0.117
fokl:							
AA	721352	0.406	293 (15.1%)	Ref.	Ref.	Ref.	Ref.
AG	2311325	0.387	895 (46.2%)	0.95 [0.84;1.09]	0.473	0.96 [0.84;1.09]	0.525
GG	1862901	0.403	750 (38.7%)	0.99 [0.86;1.13]	0.883	1.00 [0.87;1.15]	0.988
				p.trend	0.660	p.trend	0.634
taql:							
AA	1767033	0.423	747 (38.5%)	Ref.	Ref.	Ref.	Ref.
AG	2342267	0.382	895 (46.2%)	0.90 [0.82;1.00]	0.042	0.90 [0.82;0.99]	0.032

Table S2. Adjusted HRs and 95% CI for VDR Polymorphisms with rosacea in the UK Biobank study

variables	No. of Person-Years	Incident rate	Incident rosacea	Unadjusted model		Fully adjusted model[a]	
				HR (95%CI)	p-value	HR (95%CI)	p-value
GG	786278	0.376	296 (15.3%)	0.89 [0.78;1.02]	0.091	0.88 [0.77;1.00]	0.041
				p.trend	0.079	p.trend	0.049

a. The model was adjusted by gender (male/female), age (sequential), average annual household gross income (<£18,000, £18,000- £30,999, £31,000- £51,999, £52,000- £100,000, >£100,000, And "not known" or missing), season of blood collection, education (CSEs or equivalent, A levels/AS levels or equivalent, College or University degree, NVQ or HND or HNC or equivalent, O levels/GCSEs or equivalent, professional qualifications, and None of the above), race (mixed-European, white, South Asian, black and others), alcohol consumption (daily, month to week or never), Townsend Deprivation Index (continuous), BMI(kilograms per square metre, continuous), smoking status (current, previous or never), physical activity (met-minutes per week,Consecutive), time out summer (hours, consecutive), insomnia (never, sometimes, or usually), and vitamin D supplementation (yes or no).

Table S3. The joint associations of serum 25(OH)D and genetic variants in rs731236 (TaqI) with risk of rosacea.

Serum 25OHD	overall	Rosacea-free	Incident rosacea	Stratified analysis of different alleles		Base on participants with AA and severely deficient	
				HR[a]	*p-value*	HR[a]	*p-value*
**AA**	N=133,615	N=132,868	N=747				
severely deficient	16637 (12.5%)	16538 (12.4%)	99 (13.3%)	Ref.	Ref.	Ref.	Ref.
moderately deficient	55161 (41.3%)	54825 (41.3%)	336 (45.0%)	0.99 [0.78;1.24]	0.904	1.00 [0.79;1.25]	0.965
insufficient	46070 (34.5%)	45844 (34.5%)	226 (30.3%)	0.80 [0.62;1.02]	0.068	0.80 [0.63;1.02]	0.070
optimal	15747 (11.8%)	15661 (11.8%)	86 (11.5%)	0.90 [0.67;1.22]	0.494	0.90 [0.67;1.20]	0.463
				p-trend	0.078	/	/
**AG**	N=177,117	N=176,222	N=895				
severely deficient	21443 (12.1%)	21322 (12.1%)	121 (13.5%)	Ref.	Ref.	0.93 [0.72;1.22]	0.607
moderately deficient	73193 (41.3%)	72800 (41.3%)	393 (43.9%)	0.95 [0.77;1.16]	0.603	0.87 [0.70;1.09]	0.225
insufficient	61479 (34.7%)	61204 (34.7%)	275 (30.7%)	0.80 [0.64;1.00]	0.046	0.73 [0.58;0.92]	0.008
optimal	21002 (11.9%)	20896 (11.9%)	106 (11.8%)	0.91 [0.69;1.19]	0.478	0.83 [0.63;1.10]	0.194

Table S3. The joint associations of serum 25(OH)D and genetic variants in rs731236 (TaqI) with risk of rosacea.

Serum 25OHD	overall	Rosacea-free	Incident rosacea	Stratified analysis of different alleles		Base on participants with AA and severely deficient	
				HR[a]	*p-value*	HR[a]	*p-value*
				p-trend	0.109	/	/
**GG**	N=59,477	N=59,181	N=296				
severely deficient	7128 (12.0%)	7086 (12.0%)	42 (14.2%)	Ref.	Ref.	0.96 [0.67;1.38]	0.838
moderately deficient	24819 (41.7%)	24697 (41.7%)	122 (41.2%)	0.81 [0.56;1.15]	0.234	0.80 [0.61;1.04]	0.094
insufficient	20444 (34.4%)	20334 (34.4%)	110 (37.2%)	0.87 [0.60;1.25]	0.447	0.88 [0.67;1.15]	0.348
optimal	7086 (11.9%)	7064 (11.9%)	22 (7.43%)	0.49 [0.29;0.84]	0.010	0.51 [0.32;0.81]	0.004
				p-trend	0.041	*p-trend*	0.005

a.HR was adjusted by gender (male/female), age (sequential), average annual household gross income (<£18,000, £18,000- £30,999, £31,000- £51,999, £52,000- £100,000, >£100,000, And "not known" or missing), season of blood collection, education (CSEs or equivalent, A levels/AS levels or equivalent, College or University degree, NVQ or HND or HNC or equivalent, O levels/GCSEs or equivalent, professional qualifications, and None of the above), race (mixed-European, white, South Asian, black and others), alcohol consumption (daily, month to week or never), Townsend Deprivation Index (continuous), BMI(kilograms per square metre, continuous), smoking status (current, previous or never), physical activity (met-minutes per week, Consecutive), time out summer (hours, consecutive), insomnia (never, sometimes, or usually), and vitamin D supplementation (yes or no).

Table S4. Interaction effect between serum 25OHD and sex, age, body mass index, sleeplessness, 25OHD supplement, and VDR Polymorphisms

Characteristic	HR[a]	95% CI	*p-value*
**log(25OHD) * BMI**	1.00	0.99, 1.01	0.845
**log(25OHD) * Age_at_recruitment**	1.00	0.99, 1.01	0.632
**log(25OHD) * sex**	0.86	0.75, 0.99	0.032
**log(25OHD) * time_out_summer**	1.01	0.98, 1.04	0.435
**log(25OHD) * sleeplessness**			0.360
log(25OHD) * sometimes	1.04	0.87, 1.23	
log(25OHD) * usually	1.13	0.94, 1.36	
**log(25OHD) *25OHD supplement**	1.05	0.86, 1.27	0.626
**log(25OHD) * physical activity level**	1.00	1.00, 1.00	0.210
**log(25OHD) * taql**			0.859
log(25OHD) * AG	1.01	0.51, 1.97	
log(25OHD) * AA	0.87	0.39, 1.98	
**log(25OHD) * apal**			0.587

Table S4. Interaction effect between serum 25OHD and sex, age, body mass index, sleeplessness, 25OHD supplement, and VDR Polymorphisms

**Characteristic**	**HR[a]**	**95% CI**	***p-value***
log(25OHD) * AC	0.90	0.72, 1.12	
log(25OHD) * CC	0.96	0.71, 1.29	
**log(25OHD) * bsml**			0.874
log(25OHD) * TC	1.18	0.60, 2.32	
log(25OHD) * CC	1.24	0.54, 2.86	
**log(25OHD) * fokl**			0.344
log(25OHD) * AG	1.10	0.95, 1.26	
log(25OHD) * AA	1.13	0.92, 1.37	

a.The model was adjusted by age (sequential), average annual household gross income (<£18,000, £18,000- £30,999, £31,000- £51,999, £52,000- £100,000, >£100,000, And "not known" or missing), season of blood collection, education (CSEs or equivalent, A levels/AS levels or equivalent, College or University degree, NVQ or HND or HNC or equivalent, O levels/GCSEs or equivalent, professional qualifications, and None of the above), race (mixed-European, white, South Asian, black and others), alcohol consumption (daily, month to week or never), Townsend Deprivation Index (continuous), BMI(kilograms per square metre, continuous), smoking status (current, previous or never), physical activity (met-minutes per week, Consecutive), time out summer (hours, consecutive), insomnia (never, sometimes, or usually), and vitamin D supplementation (yes or no).

Table S5. Stratified analyses of the associations between serum 25(OH)D concentrations and rosacea by sex, age, BMI, sleeplessness, Physical activity level, and 25OHD supplement.

Variable	Serum 25(OH)D concentrations (nmol/L)				*p-trend*
	severely deficient	moderately deficient	insufficient	optimal	
**sex**					
male	117 (14.5%)	375 (46.5%)	235 (29.1%)	80(9.9%)	
	Ref.	0.92[0.74;1.13]	0.68 [0.54;0.86]	0.68 [0.51;0.92]	<0.001
female	145 (12.8%)	476 (42.1%)	376 (33.3%)	134(11.8%)	
	Ref.	0.96 [0.80;1.16]	0.91 [0.75;1.11]	0.95 [0.75;1.22]	0.803
**Age_at_recruitment, years**					
young	177 (15.9%)	494 (44.4%)	336 (30.2%)	106 (9.52%)	
	Ref.	0.89 [0.75;1.07]	0.80 [0.67;0.97]	0.74 [0.57;0.95]	0.047
old	85 (10.3%)	357 (43.3%)	275 (33.3%)	108 (13.1%)	
	Ref.	1.02 [0.80;1.29]	0.82 [0.64;1.06]	0.96 [0.72;1.29]	0.069
**BMI, kg/m^2**					
< 30	161 (11.3%)	605 (42.4%)	482 (33.8%)	178 (12.5%)	

Table S5. Stratified analyses of the associations between serum 25(OH)D concentrations and rosacea by sex, age, BMI, sleeplessness, Physical activity level, and 25OHD supplement.

Variable	Serum 25(OH)D concentrations (nmol/L)				*p-trend*
	**severely deficient**	**moderately deficient**	**insufficient**	**optimal**	
	Ref.	0.99 [0.83;1.18]	0.84 [0.69;1.01]	0.83 [0.67;1.04]	0.022
≥30	101 (19.7%)	246 (48.0%)	129 (25.2%)	36 (7.03%)	
	Ref.	0.87 [0.68;1.09]	0.77 [0.59;1.01]	0.94 [0.64;1.39]	0.291
**Physical activity level**					
<120 MET minutes/week	252 (13.9%)	798 (44.1%)	569 (31.5%)	189 (10.5%)	
	Ref.	0.92 [0.80;1.07]	0.80 [0.69;0.93]	0.79 [0.65;0.97]	0.008
≥120 MET-minutes/week	10 (7.69%)	53 (40.8%)	42 (32.3%)	25 (19.2%)	
	Ref.	1.37 [0.69;2.73]	1.05 [0.51;2.16]	1.56 [0.72;3.36]	0.342
**Sleeplessness**					
never	59 (13.8%)	186 (43.7%)	144 (33.8%)	37 (8.69%)	
	Ref.	0.90 [0.67;1.21]	0.83 [0.60;1.14]	0.62 [0.40;0.95]	0.120
sometimes	121 (13.3%)	415 (45.7%)	276 (30.4%)	97 (10.7%)	

Table S5. Stratified analyses of the associations between serum 25(OH)D concentrations and rosacea by sex, age, BMI, sleeplessness, Physical activity level, and 25OHD supplement.

Variable	Serum 25(OH)D concentrations (nmol/L)				*p-trend*
	severely deficient	moderately deficient	insufficient	optimal	
	Ref.	0.97 [0.79;1.19]	0.78 [0.62;0.97]	0.83 [0.63;1.09]	0.021
usually	82 (13.6%)	250 (41.5%)	191 (31.7%)	80 (13.3%)	
	Ref.	0.92 [0.72;1.19]	0.85 [0.65;1.11]	1.01 [0.73;1.39]	0.492
**25OHD supplement**					
yes	23 (6.63%)	116 (33.4%)	146 (42.1%)	62 (17.9%)	
	Ref.	0.82 [0.52;1.29]	0.81 [0.51;1.27]	0.86 [0.52;1.42]	0.805
no	239 (15.0%)	735 (46.2%)	465 (29.2%)	152 (9.55%)	
	Ref.	0.96 [0.83;1.12]	0.81 [0.69;0.95]	0.81 [0.66;1.00]	0.007

a.HR was adjusted by gender (male/female), age (sequential), average annual household gross income (<£18,000, £18,000- £30,999, £31,000- £51,999, £52,000- £100,000, >£100,000, And "not known" or missing), season of blood collection, education (CSEs or equivalent, A levels/AS levels or equivalent, College or University degree, NVQ or HND or HNC or equivalent, O levels/GCSEs or equivalent, professional qualifications, and None of the above), race (mixed-European, white, South Asian, black and others), alcohol consumption (daily, month to week or never), Townsend Deprivation Index (continuous), BMI(kilograms per square metre, continuous), smoking status (current, previous or never), physical activity (met-minutes per week,Consecutive), time out summer (hours, consecutive), insomnia (never, sometimes, or usually), and vitamin D supplementation (yes or no). The strata variable was not included in the model when stratifying by itself.

Table S6. Multivariable-adjusted HRs (95% CIs) for associations between serum 25OHD concentrations and rosacea according to different sex among participants younger than 60 years old.

Serum 25OHD	Male					Female				
	No. of Person-Years	Incident rate	Incident rosacea	Fully adjusted model[a]		No. of Person-Years	Incident rate	Incident rosacea	Fully adjusted model[a]	
				HR(95%CI)	*P*				HR(95%CI)	*P*
25OHD (nmol/L)										
severely deficient	183471	0.39	72 (18.5%)	ref	ref	212773	0.49	105 (14.5%)	ref	ref
moderately deficient	551434	0.34	188 (48.2%)	0.86 [0.64;1.10]	0.274	645811	0.47	306 (42.3%)	0.93 [0.74;1.16]	0.509
insufficient	392608	0.25	98 (25.1%)	0.63 [0.46;0.86]	0.004	496493	0.48	238 (32.9%)	0.92 [0.73;1.17]	0.503
Optimal	129110	0.25	32(8.2%)	0.63 [0.41;0.98]	0.038	175074	0.42	74 (10.3%)	0.81 [0.59;1.10]	0.179
				p-trend	0.012				p-trend	0.602
25OHD quantile (nmol/L)										
Q1 (< 33.5)	363706	0.38	139 (35.6%)	ref	ref	423241	0.50	211 (29.2%)	ref	ref
Q2 (33.5 to 48.0)	326078	0.33	108 (27.7%)	0.85 [0.66;1.10]	0.229	383794	0.45	174 (24.1%)	0.89 [0.72;1.09]	0.246

Table S6. Multivariable-adjusted HRs (95% CIs) for associations between serum 25OHD concentrations and rosacea according to different sex among participants younger than 60 years old.

Serum 25OHD	Male					Female				
	No. of Person-Years	Incident rate	Incident rosacea	Fully adjusted model[a]		No. of Person-Years	Incident rate	Incident rosacea	Fully adjusted model[a]	
				HR(95%CI)	*P*				HR(95%CI)	*P*
Q3 (48.0 to 63.4)	296448	0.27	79 (20.3%)	0.69 [0.52;0.92]	0.010	361668	0.48	174 (24.1%)	0.93 [0.76;1.14]	0.485
Q4 (> 63.4)	270390	0.24	64 (16.4%)	0.62 [0.45;0.84]	0.002	361449	0.45	164 (22.6%)	0.87 [0.70;1.08]	0.207
				p-trend	0.008				p-trend	0.570
Log(25OHD), per SD (nmol/L)	1256622	0.31	390	0.79 [0.68;0.92]	0.002	1530152	0.47	723	0.92 [0.82;1.03]	0.147

a.The model was adjusted by average annual household gross income (<£18,000, £18,000- £30,999, £31,000- £51,999, £52,000- £100,000, >£100,000, And "not known" or missing), season of blood collection, education (CSEs or equivalent, A levels/AS levels or equivalent, College or University degree, NVQ or HND or HNC or equivalent, O levels/GCSEs or equivalent, professional qualifications, and None of the above), race (mixed-European, white, South Asian, black and others), alcohol consumption (daily, month to week or never), Townsend Deprivation Index (continuous), BMI(kilograms per square metre, continuous), smoking status (current, previous or never), physical activity (met-minutes per week,Consecutive), time out summer (hours, consecutive), insomnia (never, sometimes, or usually), and vitamin D supplementation (yes or no).

Table S7. Multivariable-adjusted HRs (95% CIs) for associations between VDR Polymorphisms and rosacea according to different sex.

VDR Polymorphisms	male					Female				
	No. of Person-Years	Incident rate	Incident rosacea	Fully adjusted modela		No. of Person-Years	Incident rate	Incidnt rosacea	Fully adjusted modela	
				HR(95%CI)	*P*				HR(95%CI)	*P*
apal, n (%)										
CC	1549055	0.38	195 (24.2%)	ref	ref	581104	0.45	264 (23.3%)	ref	ref
AC	1142464	0.35	405 (50.2%)	0.93 [0.78;1.10]	0.399	1296329	0.43	560 (49.5%)	0.95 [0.82;1.10]	0.524
AA	639448	0.32	207 (25.7%)	0.85 [0.70;1.03]	0.099	725646	0.42	307 (27.1%)	0.94 [0.80;1.11]	0.455
				p-trend	0.253				p-trend	0.738
bsml, n (%)										
CC	807579	0.37	300 (37.2%)	ref	ref	922263	0.46	426 (37.7%)	ref	ref
TC	1104555	0.35	381 (47.2%)	0.93 [0.80;1.08]	0.326	1251092	0.42	526 (46.5%)	0.90 [0.80;1.03]	0.121
TT	380365	0.33	126 (15.6%)	0.89 [0.72;1.09]	0.269	429724	0.42	179 (15.8%)	0.89 [0.75;1.07]	0.211
				p-trend	0.459				p-trend	0.242
fokl, n (%)										

Table S7. Multivariable-adjusted HRs (95% CIs) for associations between VDR Polymorphisms and rosacea according to different sex.

VDR Polymorphisms	male					Female				
	No. of Person-Years	Incident rate	Incident rosacea	Fully adjusted modela		No. of Person-Years	Incident rate	Incidnt rosacea	Fully adjusted modela	
				HR(95%CI)	*P*				HR(95%CI)	*P*
AA	337669	0.39	131 (16.2%)	ref	ref	383683	0.42	162 (14.3%)	ref	ref
AG	1085115	0.32	351 (43.5%)	0.83 [0.68;1.02]	0.075	1226210	0.44	544 (48.1%)	1.06 [0.89;1.26]	0.524
GG	869714	0.37	325 (40.3%)	0.96 [0.79;1.18]	0.714	993186	0.43	425 (37.6%)	1.03 [0.86;1.23]	0.754
				p-trend	0.086				p-trend	0.791
taql, n (%)										
AA	825356	0.38	314 (38.9%)	ref	ref	941677	0.46	433 (38.3%)	ref	ref
AG	1098562	0.34	374 (46.3%)	0.89 [0.77;1.04]	0.142	1243705	0.42	521 (46.1%)	0.90 [0.79;1.03]	0.116
GG	368581	0.32	119 (14.7%)	0.85 [0.69;1.05]	0.124	417697	0.42	177 (15.6%)	0.91 [0.76;1.08]	0.291
				p-trend	0.197				p-trend	0.263

a.The model was adjusted by age (sequential), average annual household gross income (<£18,000, £18,000- £30,999, £31,000- £51,999, £52,000-£100,000, >£100,000, And "not known" or missing), season of blood collection, education (CSEs or equivalent, A levels/AS levels or equivalent, College or University degree, NVQ or HND or HNC or equivalent, O levels/GCSEs or equivalent, professional qualifications, and None of the above), race (mixed-European,

white, South Asian, black and others), alcohol consumption (daily, month to week or never), Townsend Deprivation Index (continuous), BMI(kilograms per square metre, continuous), smoking status (current, previous or never), physical activity (met-minutes per week,Consecutive), time out summer (hours, consecutive), insomnia (never, sometimes, or usually), and vitamin D supplementation (yes or no).

Table S8. Association of serum 25OHD with incident rosacea in participants with male and with GG allele of rs731236 (*TaqI*).

variables	overall *N=28095*	Rosacea-free *N=27976*	Incident rosacea *N=119*	*HR*[a]	*p-value*	*p.trend*
serum_25OHD:						0.260
severely deficient	3325 (11.8%)	3304 (11.8%)	21 (17.6%)	Ref.	Ref.	
moderately deficient	11818 (42.1%)	11771 (42.1%)	47 (39.5%)	0.62 [0.36;1.05]	0.076	
insufficient	9643 (34.3%)	9603 (34.3%)	40 (33.6%)	0.62 [0.35;1.09]	0.100	
optimal	3309 (11.8%)	3298 (11.8%)	11 (9.24%)	0.50 [0.23;1.06]	0.072	
25OHD_quantile:						0.527
Q1 (< 33.5)	6957 (24.8%)	6921 (24.7%)	36 (30.3%)	Ref.	Ref.	
Q2 (33.5 to 48.0)	7173 (25.5%)	7144 (25.5%)	29 (24.4%)	0.76 [0.46;1.26]	0.291	
Q3 (48.0 to 63.4)	7029 (25.0%)	7001 (25.0%)	28 (23.5%)	0.74 [0.44;1.24]	0.251	
Q4 (> 63.4)	6936 (24.7%)	6910 (24.7%)	26 (21.8%)	0.69 [0.40;1.17]	0.170	
log(25OHD)	5.49 (0.66)	5.49 (0.66)	5.38 (0.75)	0.76 [0.57;1.00]	0.051	0.051

a.The model was adjusted by age (sequential), average annual household gross income (<£18,000, £18,000- £30,999, £31,000- £51,999, £52,000- £100,000, >£100,000, And "not known" or missing), season of blood collection, education (CSEs or equivalent, A levels/AS levels or equivalent,

College or University degree, NVQ or HND or HNC or equivalent, O levels/GCSEs or equivalent, professional qualifications, and None of the above), race (mixed-European, white, South Asian, black and others), alcohol consumption (daily, month to week or never), Townsend Deprivation Index (continuous), BMI(kilograms per square metre, continuous), smoking status (current, previous or never), physical activity (met-minutes per week,Consecutive), time out summer (hours, consecutive), insomnia (never, sometimes, or usually), and vitamin D supplementation (yes or no).

Table S9. Sensitivity analysis for exclusion criteria

variables	excluding outcomes occurred within 2 years of follow up		further adjustment of vitamin D supplements		further adjustment of sleeplessness status	
	HR[a] (95%CI)	p-value	HR[a] (95%CI)	p-value	HR[a] (95%CI)	p-value
Serum 25OHD:						
severely deficient	Ref.	Ref.	Ref.	Ref.	Ref.	Ref.
moderately deficient	0.91 [0.78;1.06]	0.225	0.94 [0.80;1.10]	0.426	0.92 [0.76;1.11]	0.385
insufficient	0.82 [0.70;0.97]	0.018	0.81 [0.68;0.97]	0.020	0.78 [0.63;0.96]	0.020
optimal	0.76 [0.61;0.93]	0.009	0.71 [0.56;0.90]	0.005	0.67 [0.51;0.90]	0.007
	p.trend	0.024	p.trend	0.006	p.trend	0.009
25OHD_quantile:						
Q1 (< 33.5)	Ref.	Ref.	Ref.	Ref.	Ref.	Ref.
Q2 (33.5 to 48.0)	0.88 [0.76;1.00]	0.055	0.90 [0.78;1.04]	0.168	0.83 [0.70;0.99]	0.035
Q3 (48.0 to 63.4)	0.86 [0.75;0.99]	0.038	0.86 [0.74;1.00]	0.044	0.80 [0.66;0.95]	0.013
Q4 (> 63.4)	0.77 [0.66;0.89]	<0.001	0.74 [0.63;0.87]	<0.001	0.71 [0.59;0.86]	<0.001
	p.trend	0.005	p.trend	0.003	p.trend	0.004
log(25OHD)	0.87 [0.81;0.94]	<0.001	0.86 [0.79;0.94]	<0.001	0.84 [0.76;0.93]	<0.001

a.The model was adjusted by gender (male/female), age (sequential), average annual household gross income (<£18,000, £18,000- £30,999, £31,000- £51,999, £52,000- £100,000, >£100,000, And "not known" or missing), season of blood collection, education (CSEs or equivalent, A levels/AS levels or equivalent, College or University degree, NVQ or HND or HNC or equivalent, O levels/GCSEs or equivalent, professional qualifications, and None of the above), race (mixed-European, white, South Asian, black and others), alcohol consumption (daily, month to week or never), Townsend Deprivation Index (continuous), BMI(kilograms per square metre, continuous), smoking status (current, previous or never), physical activity (met-minutes per week,Consecutive), time out summer (hours, consecutive), insomnia (never, sometimes, or usually), and vitamin D supplementation (yes or no). The strata variable was not included in the model when adjusted by itself.

Table S10. Instrumental variables used in MR analysis of the association between Serum 25-Hydroxyvitamin D levels and rosacea.

id.exposure	SNP	chr	pos	alt	ref	beta	eaf	se	pval	R2
ebi-a-GCST90000615	rs10028494	4	69970937	C	A	0.028	0.225	0.002	5.18E-33	2.20E-04
ebi-a-GCST90000615	rs10084913	4	72338030	G	A	0.019	0.134	0.003	8.73E-11	9.35E-05
ebi-a-GCST90000615	rs1047891	2	211540507	A	C	-0.013	0.316	0.002	2.58E-09	6.90E-05
ebi-a-GCST90000615	rs10766281	11	15728444	G	A	0.015	0.256	0.002	7.62E-12	9.53E-05
ebi-a-GCST90000615	rs10822145	10	64934548	T	C	-0.013	0.475	0.002	1.60E-10	7.90E-05
ebi-a-GCST90000615	rs10859995	12	96375682	C	T	-0.040	0.583	0.002	1.11E-89	7.13E-04
ebi-a-GCST90000615	rs10908469	1	155509107	C	A	0.016	0.270	0.002	6.67E-13	1.03E-04
ebi-a-GCST90000615	rs11076175	16	57006378	G	A	0.024	0.178	0.003	9.46E-21	1.64E-04
ebi-a-GCST90000615	rs11182428	12	38526387	C	T	-0.012	0.520	0.002	3.17E-10	7.61E-05
ebi-a-GCST90000615	rs113642272	3	85581386	T	G	-0.020	0.636	0.002	1.73E-21	1.56E-04
ebi-a-GCST90000615	rs11458206	3	125125159	TG	T	0.014	0.272	0.002	1.18E-09	8.11E-05
ebi-a-GCST90000615	rs11542462	16	82033810	A	G	-0.023	0.134	0.003	5.79E-16	9.18E-05
ebi-a-GCST90000615	rs115621755	22	50853134	T	C	-0.013	0.327	0.002	1.53E-09	7.68E-05
ebi-a-GCST90000615	rs11606	19	54658102	G	C	0.011	0.425	0.002	2.77E-08	5.82E-05
ebi-a-GCST90000615	rs116970203	11	14876718	A	G	-0.377	0.027	0.006	1.00E-200	5.46E-03
ebi-a-GCST90000615	rs11751024	6	32586236	A	C	-0.013	0.397	0.002	3.76E-10	7.56E-05
ebi-a-GCST90000615	rs12056768	8	116988527	G	T	-0.022	0.583	0.002	3.27E-28	2.40E-04
ebi-a-GCST90000615	rs12123821	1	152179152	T	C	0.077	0.048	0.005	3.71E-63	4.54E-04
ebi-a-GCST90000615	rs12317268	12	21352541	G	A	-0.021	0.151	0.003	5.27E-14	1.72E-04
ebi-a-GCST90000615	rs12462826	19	11955767	A	G	-0.013	0.369	0.002	1.52E-10	7.62E-05
ebi-a-GCST90000615	rs1260326	2	27730940	C	T	0.022	0.607	0.002	3.95E-29	2.30E-04
ebi-a-GCST90000615	rs12798050	11	71223256	T	C	0.111	0.830	0.003	1.00E-200	5.38E-03
ebi-a-GCST90000615	rs12949853	17	7570878	A	G	0.014	0.807	0.003	1.23E-08	6.04E-05
ebi-a-GCST90000615	rs13011615	2	21274167	T	A	0.017	0.135	0.003	4.04E-09	6.89E-05
ebi-a-GCST90000615	rs13284054	9	107669073	C	T	0.019	0.118	0.003	1.82E-09	6.76E-05

ebi-a-GCST90000615	rs1352846	4	72617775	G	A	-0.193	0.291	0.002	1.00E-200	1.24E-02
ebi-a-GCST90000615	rs142004400	14	50829560	C	A	-0.033	0.035	0.005	6.43E-10	4.46E-05
ebi-a-GCST90000615	rs142158911	19	11190534	A	G	0.027	0.115	0.003	7.17E-18	1.31E-04
ebi-a-GCST90000615	rs143069752	6	40962537	A	T	0.022	0.067	0.004	1.16E-08	5.63E-05
ebi-a-GCST90000615	rs1532085	15	58683366	G	A	0.026	0.615	0.002	3.62E-38	3.30E-04
ebi-a-GCST90000615	rs1660818	11	71028249	A	G	0.016	0.323	0.002	2.74E-14	1.05E-04
ebi-a-GCST90000615	rs17144574	7	21563471	C	T	-0.015	0.234	0.002	6.73E-11	8.08E-05
ebi-a-GCST90000615	rs1800588	15	58723675	T	C	-0.033	0.215	0.002	1.50E-43	4.06E-04
ebi-a-GCST90000615	rs1841850	20	52718555	C	A	0.028	0.113	0.003	2.31E-19	2.36E-04
ebi-a-GCST90000615	rs1858889	7	107117447	C	A	0.011	0.502	0.002	1.38E-08	6.16E-05
ebi-a-GCST90000615	rs189407772	4	100146674	G	A	0.053	0.023	0.007	1.02E-15	3.08E-05
ebi-a-GCST90000615	rs1966478	5	118627319	C	T	-0.012	0.693	0.002	6.92E-09	6.66E-05
ebi-a-GCST90000615	rs2012736	2	234622379	A	C	-0.048	0.081	0.004	1.10E-40	3.18E-04
ebi-a-GCST90000615	rs2037511	18	61366207	A	G	0.017	0.166	0.003	1.07E-10	7.83E-05
ebi-a-GCST90000615	rs2074735	22	31535872	C	G	0.028	0.064	0.004	4.00E-12	1.89E-04
ebi-a-GCST90000615	rs212100	19	48376995	C	T	-0.066	0.836	0.003	7.83E-136	1.11E-03
ebi-a-GCST90000615	rs2123930	15	100231033	A	G	-0.014	0.279	0.002	1.30E-10	8.65E-05
ebi-a-GCST90000615	rs2131925	1	63025942	T	G	-0.022	0.644	0.002	2.43E-27	1.92E-04
ebi-a-GCST90000615	rs2171427	12	24822366	A	G	-0.015	0.155	0.003	1.81E-08	6.24E-05
ebi-a-GCST90000615	rs2207132	20	39142516	A	G	-0.039	0.033	0.005	1.96E-12	1.82E-04
ebi-a-GCST90000615	rs2229742	21	16339172	C	G	-0.025	0.103	0.003	5.90E-15	1.25E-04
ebi-a-GCST90000615	rs2246832	3	49881134	T	A	0.017	0.501	0.002	1.87E-18	1.37E-04
ebi-a-GCST90000615	rs2276360	11	71169547	C	G	0.111	0.788	0.002	1.00E-200	5.87E-03
ebi-a-GCST90000615	rs2297991	10	113913222	C	T	0.012	0.721	0.002	3.51E-08	6.49E-05
ebi-a-GCST90000615	rs2346264	7	133536351	C	A	-0.015	0.783	0.002	8.86E-10	5.72E-05
ebi-a-GCST90000615	rs2579309	4	72077830	A	G	0.023	0.067	0.004	6.23E-09	5.83E-05
ebi-a-GCST90000615	rs2608984	6	131918839	T	A	-0.021	0.165	0.003	2.79E-15	1.46E-04
ebi-a-GCST90000615	rs2642439	1	220970499	G	A	-0.015	0.685	0.002	6.18E-12	8.66E-05

ebi-a-GCST90000615	rs2659007	17	79217478	A	G	0.011	0.451	0.002	9.75E-09	6.43E-05
ebi-a-GCST90000615	rs2756119	14	104001517	A	G	0.012	0.383	0.002	2.35E-09	7.02E-05
ebi-a-GCST90000615	rs2847500	11	120114421	A	G	-0.022	0.124	0.003	1.22E-13	1.24E-04
ebi-a-GCST90000615	rs2952289	17	66464414	T	C	0.018	0.798	0.002	3.22E-13	9.39E-05
ebi-a-GCST90000615	rs33981819	11	66079361	G	T	0.011	0.461	0.002	1.20E-08	6.30E-05
ebi-a-GCST90000615	rs34177108	16	89893375	A	C	-0.012	0.268	0.002	3.96E-08	4.84E-05
ebi-a-GCST90000615	rs34186890	3	141720712	G	A	-0.015	0.260	0.002	2.25E-11	8.33E-05
ebi-a-GCST90000615	rs34726834	8	25889606	T	C	0.015	0.252	0.002	4.49E-11	9.34E-05
ebi-a-GCST90000615	rs35408430	1	17560195	T	C	-0.021	0.342	0.002	5.82E-25	2.12E-04
ebi-a-GCST90000615	rs36037728	11	71111987	T	C	-0.059	0.026	0.006	7.69E-22	1.29E-04
ebi-a-GCST90000615	rs36102036	4	72912236	A	G	0.034	0.345	0.002	8.71E-60	5.16E-04
ebi-a-GCST90000615	rs3814995	19	36342212	T	C	-0.013	0.312	0.002	2.33E-09	7.38E-05
ebi-a-GCST90000615	rs3890624	4	166251068	G	A	0.011	0.395	0.002	3.19E-08	5.78E-05
ebi-a-GCST90000615	rs3925446	10	91524012	A	G	0.016	0.199	0.002	3.68E-11	1.10E-04
ebi-a-GCST90000615	rs4121823	18	47144223	A	T	-0.019	0.845	0.003	1.33E-11	9.26E-05
ebi-a-GCST90000615	rs41301394	7	75612803	T	C	0.013	0.282	0.002	7.11E-09	7.55E-05
ebi-a-GCST90000615	rs429358	19	45411941	C	T	-0.023	0.152	0.003	1.76E-17	1.61E-04
ebi-a-GCST90000615	rs4364259	4	15892159	A	G	0.017	0.202	0.002	2.23E-11	9.38E-05
ebi-a-GCST90000615	rs4418728	10	94839724	T	G	0.012	0.452	0.002	7.54E-10	7.32E-05
ebi-a-GCST90000615	rs4616820	4	57745481	T	C	-0.012	0.465	0.002	5.32E-10	7.51E-05
ebi-a-GCST90000615	rs512083	1	46027355	C	T	0.012	0.461	0.002	6.56E-10	7.44E-05
ebi-a-GCST90000615	rs532436	9	136149830	A	G	-0.017	0.184	0.003	1.82E-11	9.27E-05
ebi-a-GCST90000615	rs57459725	8	61312205	G	C	-0.018	0.133	0.003	1.07E-09	8.86E-05
ebi-a-GCST90000615	rs575976	11	75437630	G	A	0.016	0.182	0.003	1.06E-09	1.03E-04
ebi-a-GCST90000615	rs58387006	2	32579999	C	A	-0.014	0.222	0.002	6.33E-09	6.68E-05
ebi-a-GCST90000615	rs5965373	X	66409433	G	T	0.013	0.857	0.002	1.41E-08	4.72E-05
ebi-a-GCST90000615	rs6003465	22	23365501	C	T	-0.012	0.332	0.002	1.19E-08	5.62E-05
ebi-a-GCST90000615	rs61003750	17	40812065	C	G	-0.012	0.307	0.002	1.85E-08	5.76E-05

ebi-a-GCST90000615	rs61816766	1	152319572	C	T	0.087	0.029	0.006	2.75E-49	1.18E-04
ebi-a-GCST90000615	rs62012775	15	63871274	T	A	-0.016	0.176	0.003	2.77E-10	9.45E-05
ebi-a-GCST90000615	rs62115743	19	51514874	T	C	0.027	0.082	0.004	3.21E-14	1.36E-04
ebi-a-GCST90000615	rs6547409	2	21190209	T	C	0.028	0.050	0.005	6.57E-10	1.11E-04
ebi-a-GCST90000615	rs6671730	1	2339139	A	G	-0.015	0.434	0.002	2.25E-14	1.14E-04
ebi-a-GCST90000615	rs6672758	1	230303512	T	C	0.016	0.801	0.002	7.16E-11	9.93E-05
ebi-a-GCST90000615	rs6723486	2	58979783	T	C	-0.011	0.611	0.002	4.24E-08	6.15E-05
ebi-a-GCST90000615	rs6724965	2	101440151	G	A	-0.017	0.171	0.003	1.76E-10	9.21E-05
ebi-a-GCST90000615	rs6834488	4	88178919	T	C	-0.015	0.424	0.002	1.96E-13	9.40E-05
ebi-a-GCST90000615	rs7128011	11	14462532	A	G	-0.075	0.368	0.002	1.00E-200	2.59E-03
ebi-a-GCST90000615	rs72834856	6	22801858	G	T	-0.024	0.072	0.004	4.86E-10	6.56E-05
ebi-a-GCST90000615	rs73413596	12	111582630	C	T	0.024	0.074	0.004	2.89E-10	6.15E-05
ebi-a-GCST90000615	rs7522116	1	41835685	T	C	-0.013	0.566	0.002	2.58E-10	7.61E-05
ebi-a-GCST90000615	rs7528419	1	109817192	G	A	0.020	0.225	0.002	1.34E-17	1.36E-04
ebi-a-GCST90000615	rs7569755	2	118648261	A	G	0.013	0.291	0.002	1.21E-09	5.92E-05
ebi-a-GCST90000615	rs77037130	11	14467795	A	G	-0.068	0.015	0.008	1.55E-17	1.46E-04
ebi-a-GCST90000615	rs7784802	7	64017227	T	A	0.014	0.361	0.002	1.40E-11	7.58E-05
ebi-a-GCST90000615	rs77924615	16	20392332	A	G	-0.014	0.193	0.003	8.09E-09	7.26E-05
ebi-a-GCST90000615	rs78649910	4	3482213	A	T	-0.019	0.106	0.003	1.57E-09	7.96E-05
ebi-a-GCST90000615	rs79598313	1	27284913	T	C	-0.037	0.023	0.006	1.13E-08	9.18E-05
ebi-a-GCST90000615	rs79687284	1	214150821	C	G	-0.030	0.035	0.005	3.77E-08	3.02E-05
ebi-a-GCST90000615	rs8018720	14	39556185	C	G	-0.038	0.823	0.003	1.95E-50	3.73E-04
ebi-a-GCST90000615	rs804281	8	11611865	G	A	0.016	0.584	0.002	2.48E-15	1.07E-04
ebi-a-GCST90000615	rs8063565	16	30883965	C	G	0.012	0.734	0.002	2.68E-08	6.26E-05
ebi-a-GCST90000615	rs8091117	18	28919794	A	C	-0.025	0.065	0.004	2.34E-10	9.15E-05
ebi-a-GCST90000615	rs8121940	20	52742306	G	C	-0.038	0.195	0.002	1.69E-52	5.07E-04
ebi-a-GCST90000615	rs8181687	12	68661596	A	G	0.012	0.579	0.002	4.00E-09	6.70E-05
ebi-a-GCST90000615	rs9409266	9	125745042	A	G	-0.018	0.861	0.003	8.74E-11	9.59E-05

ebi-a-GCST90000615	rs9467550	6	25653401	G	A	-0.019	0.105	0.003	3.36E-09	7.18E-05
ebi-a-GCST90000615	rs9476310	6	57767576	T	C	0.012	0.511	0.002	2.67E-09	6.80E-05
ebi-a-GCST90000615	rs964184	11	116648917	C	G	0.043	0.868	0.003	3.37E-50	4.64E-04
GCST010144	rs1011468	7	104613791	A	G	-0.014	0.476	0.042	1.35E-12	9.48E-05
GCST010144	rs10127775	1	230295789	T	A	0.012	0.605	0.042	3.43E-09	6.90E-05
GCST010144	rs10426	19	51517798	A	G	0.025	0.213	0.047	3.31E-26	2.50E-04
GCST010144	rs1047891	2	211540507	A	C	-0.014	0.316	0.044	1.16E-11	8.79E-05
GCST010144	rs10500209	19	11979164	C	T	-0.013	0.282	0.045	6.18E-10	7.54E-05
GCST010144	rs10793129	11	75459865	A	G	0.024	0.090	0.068	1.64E-12	1.13E-04
GCST010144	rs10818769	9	125719923	G	C	-0.017	0.857	0.055	3.35E-09	8.16E-05
GCST010144	rs10832218	11	14181174	C	T	-0.034	0.198	0.044	7.09E-32	5.31E-04
GCST010144	rs10832289	11	14669802	T	A	-0.069	0.410	0.042	1.00E-200	2.27E-03
GCST010144	rs10859995	12	96375682	C	T	-0.039	0.581	0.044	7.03E-89	6.94E-04
GCST010144	rs10887718	10	82042624	T	C	-0.012	0.527	0.042	1.44E-10	7.58E-05
GCST010144	rs11127048	2	27752463	A	G	0.018	0.617	0.043	6.41E-19	1.53E-04
GCST010144	rs111529171	7	21571932	C	G	-0.015	0.216	0.054	6.24E-11	7.22E-05
GCST010144	rs112285002	19	48385057	T	C	0.060	0.160	0.054	1.77E-110	1.07E-03
GCST010144	rs11264360	1	155304581	A	T	0.018	0.243	0.046	3.34E-15	1.30E-04
GCST010144	rs113938679	4	72488025	A	G	-0.184	0.006	0.167	5.88E-36	1.12E-03
GCST010144	rs1149605	11	76485216	C	T	0.019	0.171	0.056	7.34E-14	1.04E-04
GCST010144	rs115045402	1	152029548	A	G	0.107	0.026	0.128	3.05E-55	6.38E-04
GCST010144	rs11723621	4	72615362	G	A	-0.187	0.291	0.052	1.00E-200	1.12E-02
GCST010144	rs117576073	11	14912573	T	G	-0.115	0.012	0.131	1.22E-38	6.48E-04
GCST010144	rs117913124	11	14900931	A	G	-0.354	0.028	0.153	1.00E-200	4.84E-03
GCST010144	rs12123821	1	152179152	T	C	0.074	0.048	0.107	2.25E-59	4.22E-04
GCST010144	rs1229984	4	100239319	C	T	-0.047	0.973	0.292	4.85E-13	2.36E-05
GCST010144	rs12317268	12	21352541	G	A	-0.019	0.152	0.046	9.15E-12	1.39E-04
GCST010144	rs12803256	11	71132868	G	A	0.100	0.771	0.042	1.00E-200	4.92E-03

GCST010144	rs12997242	2	21383353	A	G	-0.013	0.438	0.044	2.23E-10	7.03E-05
GCST010144	rs138726443	1	152280023	A	G	0.112	0.005	0.244	8.81E-15	1.85E-04
GCST010144	rs143106299	4	72920085	T	A	-0.169	0.006	0.468	1.50E-28	1.15E-04
GCST010144	rs144613541	1	152270875	G	A	0.015	0.291	0.049	6.49E-12	8.53E-05
GCST010144	rs148514005	11	14464878	T	C	-0.447	0.006	0.729	1.37E-184	4.14E-04
GCST010144	rs157595	19	45425460	G	A	-0.016	0.614	0.045	2.95E-14	1.04E-04
GCST010144	rs17765311	15	63789952	C	A	-0.015	0.345	0.043	1.35E-13	1.09E-04
GCST010144	rs1800588	15	58723675	T	C	-0.030	0.215	0.048	2.65E-36	3.31E-04
GCST010144	rs1800775	16	56995236	A	C	-0.017	0.486	0.042	1.56E-17	1.38E-04
GCST010144	rs185433896	1	152249021	A	C	-0.246	0.993	0.897	1.50E-38	8.41E-05
GCST010144	rs1858889	7	107117447	C	A	0.013	0.501	0.042	3.85E-11	8.18E-05
GCST010144	rs188480917	11	14785870	G	C	-0.343	0.011	0.249	1.00E-200	1.68E-03
GCST010144	rs1972994	3	85631142	T	A	-0.018	0.647	0.047	7.99E-18	1.21E-04
GCST010144	rs2011425	2	234627608	G	T	-0.046	0.079	0.086	9.66E-38	2.54E-04
GCST010144	rs201501563	11	14910273	T	C	-0.066	0.122	0.043	9.17E-67	2.09E-03
GCST010144	rs2037511	18	61366207	A	G	0.016	0.165	0.057	9.29E-10	6.92E-05
GCST010144	rs2074735	22	31535872	C	G	0.027	0.064	0.060	6.55E-12	1.81E-04
GCST010144	rs222026	4	72644962	T	A	-0.052	0.871	0.052	6.98E-68	8.65E-04
GCST010144	rs2229742	21	16339172	C	G	-0.026	0.104	0.067	7.13E-16	1.31E-04
GCST010144	rs2585442	20	52737123	G	C	0.034	0.246	0.045	6.87E-49	4.91E-04
GCST010144	rs261291	15	58680178	C	T	-0.022	0.356	0.043	2.89E-28	2.39E-04
GCST010144	rs2762942	20	52788925	A	G	0.053	0.942	0.093	7.99E-35	2.87E-04
GCST010144	rs28364331	4	100201295	G	A	0.061	0.019	0.375	1.31E-17	2.41E-05
GCST010144	rs2847500	11	120114421	A	G	-0.021	0.124	0.059	7.79E-13	1.13E-04
GCST010144	rs2909218	17	66464546	T	C	0.017	0.793	0.054	2.81E-12	8.38E-05
GCST010144	rs2934744	1	63048045	A	C	-0.022	0.644	0.047	3.96E-26	1.95E-04
GCST010144	rs34726834	8	25889606	T	C	0.014	0.254	0.045	6.65E-10	7.97E-05
GCST010144	rs3750296	1	17559656	C	G	-0.021	0.341	0.043	2.09E-24	2.02E-04

GCST010144	rs3768013	1	150815411	A	G	-0.015	0.370	0.043	1.37E-13	1.01E-04
GCST010144	rs3814995	19	36342212	T	C	-0.015	0.312	0.044	2.83E-12	1.00E-04
GCST010144	rs3822868	6	131934986	G	A	0.022	0.835	0.051	1.41E-15	1.61E-04
GCST010144	rs523583	11	66070146	C	A	0.012	0.469	0.042	5.58E-10	7.40E-05
GCST010144	rs528776789	4	72486140	A	G	0.178	0.993	0.498	3.67E-31	1.26E-04
GCST010144	rs532436	9	136149830	A	G	-0.015	0.184	0.052	2.17E-09	7.27E-05
GCST010144	rs536006581	11	71135151	G	G	-0.174	0.009	0.387	8.87E-35	1.95E-04
GCST010144	rs549940584	11	71222408	T	C	0.183	0.013	0.501	2.31E-72	1.36E-04
GCST010144	rs57631352	19	4338173	G	A	-0.013	0.297	0.044	1.48E-09	7.38E-05
GCST010144	rs577185477	11	14612563	C	T	-0.379	0.015	0.195	1.00E-200	3.53E-03
GCST010144	rs58073039	4	88287363	G	A	-0.014	0.298	0.048	2.16E-11	7.64E-05
GCST010144	rs58542926	19	19379549	T	C	0.032	0.076	0.085	8.57E-19	1.27E-04
GCST010144	rs6123359	20	52714706	G	A	0.032	0.105	0.065	7.74E-24	2.12E-04
GCST010144	rs6127099	20	52731402	T	A	-0.037	0.279	0.047	9.30E-62	5.32E-04
GCST010144	rs61816761	1	152285861	A	G	0.125	0.023	0.405	8.57E-74	8.95E-05
GCST010144	rs61937878	12	96371731	T	C	0.119	0.006	0.894	4.43E-22	1.56E-05
GCST010144	rs62007299	15	77711719	A	G	-0.014	0.709	0.044	1.69E-11	9.13E-05
GCST010144	rs6438900	3	125148287	G	C	0.014	0.261	0.045	9.59E-10	7.85E-05
GCST010144	rs6698680	1	2329661	G	A	-0.012	0.464	0.042	8.99E-10	7.07E-05
GCST010144	rs6724965	2	101440151	G	A	-0.017	0.172	0.051	1.29E-10	9.17E-05
GCST010144	rs6773343	3	141825598	T	C	0.013	0.720	0.048	5.20E-09	6.19E-05
GCST010144	rs705117	4	72608115	T	C	-0.034	0.849	0.062	1.71E-36	2.66E-04
GCST010144	rs71383766	16	30945887	T	C	0.013	0.420	0.042	1.15E-09	7.77E-05
GCST010144	rs73015021	19	11192915	G	A	0.023	0.121	0.070	1.15E-14	9.49E-05
GCST010144	rs7519574	1	34726552	A	G	0.017	0.182	0.062	2.09E-11	6.56E-05
GCST010144	rs7528419	1	109817192	G	A	0.019	0.225	0.051	2.41E-16	1.22E-04
GCST010144	rs7569755	2	118648261	A	G	0.014	0.292	0.051	8.03E-11	6.55E-05
GCST010144	rs7699711	4	69947596	T	G	-0.029	0.455	0.042	6.97E-49	4.03E-04

GCST010144	rs7718395	5	118652574	G	C	0.013	0.320	0.044	1.67E-09	7.35E-05
GCST010144	rs77924615	16	20392332	A	G	-0.016	0.198	0.050	1.46E-10	8.62E-05
GCST010144	rs7828742	8	116960729	G	A	-0.022	0.597	0.042	3.06E-28	2.40E-04
GCST010144	rs78649910	4	3482213	A	T	-0.018	0.110	0.064	4.32E-09	7.16E-05
GCST010144	rs8018720	14	39556185	C	G	-0.032	0.820	0.059	4.04E-36	2.59E-04
GCST010144	rs804280	8	11612698	A	C	0.013	0.582	0.060	4.43E-11	7.34E-05
GCST010144	rs8063706	16	11909552	T	A	0.013	0.273	0.046	3.64E-09	6.88E-05
GCST010144	rs8091117	18	28919794	A	C	-0.024	0.065	0.077	1.03E-09	8.39E-05
GCST010144	rs8103262	19	53065814	C	T	0.013	0.305	0.045	3.18E-09	6.66E-05
GCST010144	rs867772	1	220972343	G	A	-0.014	0.682	0.046	3.64E-11	7.90E-05
GCST010144	rs960596	22	41393520	T	C	0.012	0.340	0.043	2.23E-09	7.17E-05
GCST010144	rs964184	11	116648917	C	G	0.040	0.864	0.059	5.11E-44	3.94E-04
GCST010144	rs9668081	12	38602911	T	C	0.012	0.471	0.042	5.38E-09	6.68E-05

Table S11. Mendelian randomization analyses for the association of serum 25OHD concentration with rosacea.

Exposure	Outcome	Number of SNPs	Method	*β*	Standard error	*P*	OR (95%CI)
Serum 25OHD concentration (id: GCST010144)	Rosacea (id: finn-b-L12_ROSACEA)	92	MR Egger	-0.43772	0.18023	0.01714	0.65 (0.45-0.92)
			Weighted median	-0.39568	0.20080	0.04878	0.67 (0.46-0.99)
			Inverse variance weighted	-0.28305	0.13681	0.03856	0.75 (0.57-0.99)
			maximum likelihood	-0.28346	0.13704	0.03859	0.75 (0.58-0.99)
			Weighted mode	-0.38646	0.17434	0.02914	0.68 (0.49-0.94)
Serum 25OHD concentration (id: ebi-a-GCST90000615)	Rosacea (id: finn-b-L12_ROSACEA)	110	MR Egger	-0.27479	0.18177	0.13352	0.76 (0.53-1.08)
			Weighted median	-0.38317	0.19933	0.05457	0.68 (0.46-1.00)
			Inverse variance weighted	-0.24673	0.13745	0.07263	0.78 (0.60-1.02)
			maximum likelihood	-0.24804	0.13777	0.07180	0.78 (0.60-1.02)
			Weighted mode	-0.31318	0.16264	0.05676	0.73 (0.53-1.01)

SNPs: single nucleotide polymorphisms.

Table S12. Test for heterogeneity in Mendelian randomization analysis.

Exposure	Outcome	Method	Q statistics	df	*Q_P*
Serum 25OHD concentration (id: GCST010144)	Rosacea (id: finn-b-L12_ROSACEA)	MR Egger	74.25096	90	0.884896
		Inverse variance weighted	75.98914	91	0.870838
Serum 25OHD concentration (id: ebi-a-GCST90000615)	Rosacea (id: finn-b-L12_ROSACEA)	MR Egger	107.9951	108	0.482034
		Inverse variance weighted	108.0507	109	0.507689

Table S13. Test for horizontal pleiotropy in Mendelian randomization analysis.

Exposure	Outcome	Egger intercept	Standard error	*P*
Serum 25OHD concentration (id: GCST010144)	Rosacea (id: finn-b-L12_ROSACEA)	0.009677	0.00734	0.190714
Serum 25OHD concentration (id: ebi-a-GCST90000615)	Rosacea (id: finn-b-L12_ROSACEA)	0.001528	0.006478	0.814001

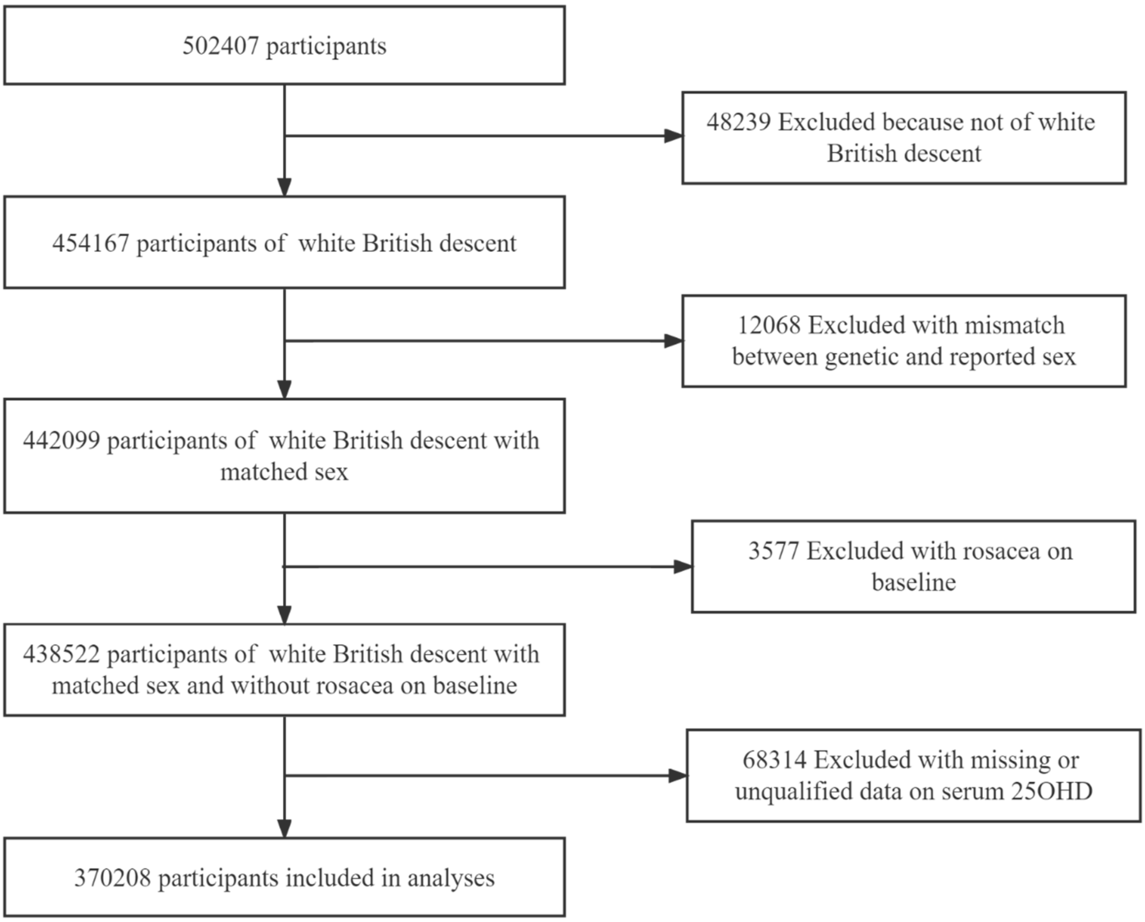

Figure S1. Flowchart for the Selection of the Analysed Study Sample from the UK Biobank Study.

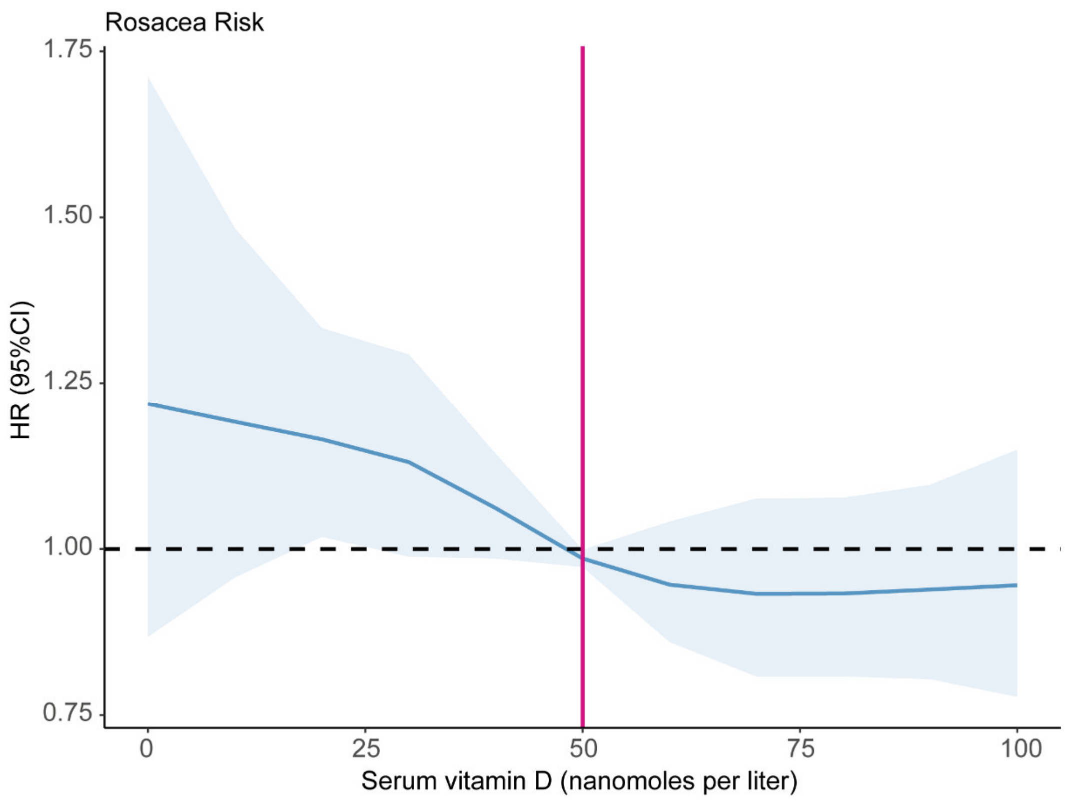

Figure S2. Associations of serum 25(OH)D concentrations with risks of rosacea in the UK Biobank. The model was adjusted by gender (male/female), age (sequential), average annual household gross income (<£18,000, £18,000- £30,999, £31,000- £51,999, £52,000- £100,000, >£100,000, And "not known" or missing), education (CSEs or equivalent, A levels/AS levels or equivalent, College or University degree, NVQ or HND or HNC or equivalent, O levels/GCSEs or equivalent, professional qualifications, and None of the above), race (mixed-European, white, South Asian, black and others), alcohol consumption (daily, month to week or never), Townsend Deprivation Index (continuous), BMI(kilograms per square metre, continuous), smoking status (current, previous or never), physical activity (met-minutes per week, Consecutive), time out summer (hours, consecutive), insomnia (never, sometimes, or usually), and vitamin D supplementation (yes or no).

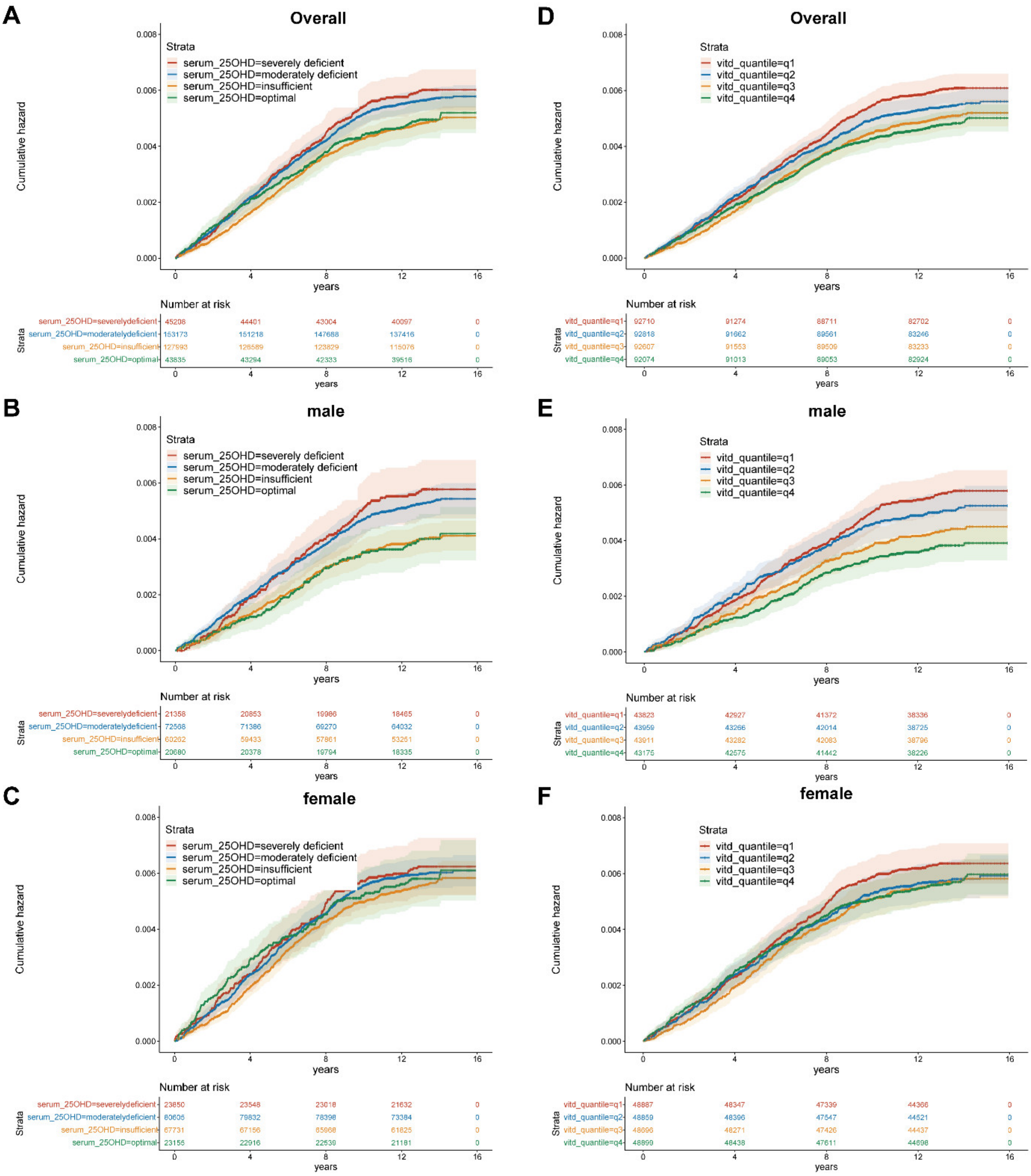

Figure S3. Cumulative risk curve for incident rosacea between different serum 25OHD categories.

Reference

1. Manousaki D, Mitchell R, Dudding T, et al. Genome-wide Association Study for Vitamin D Levels Reveals 69 Independent Loci. *Am J Hum Genet*. 2020;106(3):327-337. doi:10.1016/j.ajhg.2020.01.017
2. Kurki MI, Karjalainen J, Palta P, et al. FinnGen: Unique genetic insights from combining isolated population and national health register data. Published online March 6, 2022:2022.03.03.22271360. doi:10.1101/2022.03.03.22271360
3. Revez JA, Lin T, Qiao Z, et al. Genome-wide association study identifies 143 loci associated with 25 hydroxyvitamin D concentration. *Nat Commun*. 2020;11(1):1647. doi:10.1038/s41467-020-15421-7
4. Balduzzi S, Rücker G, Schwarzer G. How to perform a meta-analysis with R: a practical tutorial. *Evid Based Ment Health*. 2019;22(4):153-160. doi:10.1136/ebmental-2019-300117
5. Burgess S. Sample size and power calculations in Mendelian randomization with a single instrumental variable and a binary outcome. *Int J Epidemiol*. 2014;43(3):922-929. doi:10.1093/ije/dyu005
